# Identification of novel *FBN1* variations implicated in congenital scoliosis

**DOI:** 10.1038/s10038-019-0698-x

**Published:** 2019-12-11

**Authors:** Mao Lin, Sen Zhao, Gang Liu, Yingzhao Huang, Chenxi Yu, Yanxue Zhao, Lianlei Wang, Yuanqiang Zhang, Zihui Yan, Shengru Wang, Sen Liu, Jiaqi Liu, Yongyu Ye, Yaping Chen, Xu Yang, Bingdu Tong, Zheng Wang, Xinzhuang Yang, Yuchen Niu, Xiaoxin Li, Yipeng Wang, Jianzhong Su, Jian Yuan, Hengqiang Zhao, Shuyang Zhang, Guixing Qiu, on behalf of the Deciphering Disorders Involving Scoliosis and COmorbidities (DISCO) study, Guixing Qiu, Zhihong Wu, Jianguo Zhang, Nan Wu, Shengru Wang, Jiaqi Liu, Sen Liu, Yuzhi Zuo, Gang Liu, Yuanqiang Zhang, Chenxi Yu, Sen Zhao, Lianlei Wang, Yanxue Zhao, Zihui Yan, Xinzhuang Yang, Hengqiang Zhao, Yuchen Niu, Xiaoxin Li, Mao Lin, Shiro Ikegawa, Jianguo Zhang, Zhihong Wu, Nan Wu

**Affiliations:** 1 Department of Orthopedic Surgery, Peking Union Medical College Hospital, Peking Union Medical College and Chinese Academy of Medical Sciences, Beijing, 100730 China; 2 Graduate School of Peking Union Medical College, Chinese Academy of Medical Sciences, Beijing, 100005 China; 3 Laboratory for Bone and Joint Diseases, RIKEN Center for Integrative Medical Sciences, Tokyo, 108-8639 Japan; 4 Beijing Key Laboratory for Genetic Research of Skeletal Deformity, Beijing, 100730 China; 5 Department of Breast Surgical Oncology, National Cancer Center/National Clinical Research Center for Cancer/Cancer Hospital, Chinese Academy of Medical Sciences and Peking Union Medical College, Beijing, 100021 China; 6 Department of Joint Surgery, First Affiliated Hospital of Sun Yat-sen University, #58 Zhongshan 2nd Road, Guangzhou, 510080 China; 7 Department of Medical Genetics, Institute of Basic Medical Sciences, Chinese Academy of Medical Sciences and Peking Union Medical College, Beijing, 100005 China; 8 Medical Research Center & Department of Central Laboratory, Peking Union Medical College Hospital, Peking Union Medical College and Chinese Academy of Medical Sciences, Beijing, 100730 China; 9 Medical Research Center of Orthopedics, Chinese Academy of Medical Sciences, Beijing, 100730 China; 10 School of Ophthalmology & Optometry and Eye Hospital, School of Biomedical Engineering, Institute of Biomedical Big Data, Wenzhou Medical University, 325027 Wenzhou, China; 11 Department of Cardiology, Peking Union Medical College Hospital, Peking Union Medical College and Chinese Academy of Medical Sciences, No. 1 Shuaifuyuan, Beijing, 100730 China

**Keywords:** Medical genomics, Genetics research

## Abstract

Congenital scoliosis (CS) is a form of scoliosis caused by congenital vertebral malformations. Genetic predisposition has been demonstrated in CS. We previously reported that *TBX6* loss-of-function causes CS in a compound heterozygous model; however, this model can explain only 10% of CS. Many monogenic and polygenic CS genes remain to be elucidated. In this study, we analyzed exome sequencing (ES) data of 615 Chinese CS from the Deciphering Disorders Involving Scoliosis and COmorbidities (DISCO) project. Cosegregation studies for 103 familial CS identified a novel heterozygous nonsense variant, c.2649G>A (p.Trp883Ter) in *FBN1*. The association between *FBN1* and CS was then analyzed by extracting *FBN1* variants from ES data of 574 sporadic CS and 828 controls; 30 novel variants were identified and prioritized for further analyses. A mutational burden test showed that the deleterious *FBN1* variants were significantly enriched in CS subjects (OR = 3.9, *P* = 0.03 by Fisher’s exact test). One missense variant, c.2613A>C (p.Leu871Phe) was recurrent in two unrelated CS subjects, and in vitro functional experiments for the variant suggest that *FBN1* may contribute to CS by upregulating the transforming growth factor beta (TGF-β) signaling. Our study expanded the phenotypic spectrum of *FBN1*, and provided nove insights into the genetic etiology of CS.

## Introduction

Congenital scoliosis (CS) is a form of scoliosis caused by congenital vertebral malformations potentially resulting from formation failure, segmentation defects, or a combination of both [1]. The prevalence of CS is ~0.5–1 per 1000 live births [2]. As a major cause to infantile and adolescent disability, CS perturbs patient lives and daily activities both physically and psychologically [3].

Genetic predisposition has been demonstrated in CS [4, 5]. CS is a consequence of single-gene mutations. We previously reported that a heterozygous 16p11.2 deletion or rare *TBX6* loss-of-function (LoF) variants together with a common hypomorphic risk haplotype composed by three SNPs in *trans* cause CS [6]. We subsequently recapitulated this compound heterozygosity model in a gene-edited mouse [7], and defined the unique and clinically actionable phenotype of a monogenic form of CS, *TBX6*-associated CS (TACS) [8]. However, this model can explain only about 10% of CS [8, 9]. Many other contributor genes remain to be elucidated.

CS is also a consequence of multifactorial gene-environment mutual interaction [10]. The susceptibility of some polygenic defects has been illustrated in CS cases, accompanied with multiple birth defects [11]. Previous studies have reported several CS-associated genes, including *PAX1* (MIM #167411) [12], *PTK7* (MIM #601890) [13], *DLL1* (MIM #606582) [14], *DDR2* (MIM #191311) [15], *T* (MIM #601397) [16], and numerous other genes are thus far known to cause phenotypes involving scoliosis with vertebral malformations; however, their potential contribution to CS has been poorly investigated.

Here, as a part of the Deciphering Disorders Involving Scoliosis and COmorbidities (DISCO) project, we conducted exome sequencing (ES) for a CS cohort. Trio-based analyses on familial cases identified a novel nonsense variant in *FBN1*, the gene implicated in Marfan syndrome (MFS; MIM#154700) and many monogenic diseases with scoliosis and spinal dysplasia. We then examined the effect of rare *FBN1* variants on sporadic CS and observed that deleterious missense variants were significantly enriched in CS. Functional analyses of a recurrent *FBN1* missense variant revealed the potential association between upregulation of transforming growth factor beta (TGF-β) signaling and CS. The subjects carrying highly deleterious *FBN1* variants had vertebral malformations, malformations of the ribs, and intraspinal defects.

## Materials and methods

### Participant recruitment

Initially, 615 Chinese CS subjects with complete clinical data and ES data were recruited at Peking Union Medical College Hospital (PUMCH) in China from 2010 to 2018, as a pivotal part of DISCO study (http://www.discostudy.org/). There were 103 familial cases with available samples for first-degree relatives. *TBX*6 mutations were examined and 41 cases molecularly diagnosed as TACS [6, 8] were removed. Consequently, 574 CS cases were retained for further study. For the control subjects, 828 unrelated Chinese iindividuals without apparent spinal deformity were ascertained. The ethical committees or institutional review boards at PUMCH that contributed to the patient samples approved the study. Written informed consent was obtained from each participant or their guardians (for those younger than 16 years old).

### Exome Sequencing and Sanger sequencing

Genomic DNA from peripheral blood samples was extracted using the DNeasy Blood & Tissue kit (Qiagen, Hilden, Germany). The ES data were processed and analyzed using the PUMCH Pipeline as previously described [17]. The following public available databases were used for mutation annotation: the 1000 Genomes Project (1KG; http://www.internationalgenome.org), the Exome Sequencing Project (ESP; http://evs.gs.washington.edu/EVS/), Exome Aggregation Consortium (ExAC; http://exac.broadinstitute.org/), and Genome Aggregation Database (gnomAD; http://gnomad-old.broadinstitute.org), Single Nucleotide Polymorphism database (dbSNP; https://www.ncbi.nlm.nih.gov/snp). In silico prediction tools, including Sorting Intolerant From Tolerant (SIFT) [18], Polymorphism Phenotyping v2 (Polyphen-2) [19], Mutation Taster [20], and Combined Annotation Dependent Depletion (CADD) [21] were utilized to predict deleterious properties of variants. We also annotated the detected variants using customized database based on Human Gene Mutation Database (HGMD; www.hgmd.cf.ac.uk/) and Online Mendelian Inheritance in Man (https://omim.org/). Empirically, we utilized the recommended filters of the Genome Analysis Toolkit (v2.2–3) prior to performing association analyses in order to guarantee that the risk of false negatives is minimized to select quality control passed variants [22]. Sanger sequencing by using an Applied Biosystem 3730xl DNA Analyzer (Life Technologies, CA, USA) was conducted to confirm the candidate variants identified by ES, and segregation analyses were performed in family members. The RefSeq accession numbers of the transcript and corresponding protein isoform of *FBN1* we used for mutation nomenclature were NM_000138.4 and NP_000129.3, respectively.

### Mutational burden analyses

Mutational burden analyses of *FBN1* were implemented between 574 CS cases and 828 controls. To alleviate the biased factors attributable to differential sequencing coverage, we conducted harmonization analyses between case and control exomes. An individual RefSeq coding sequence site was excluded from the analysis if the absolute difference in percentages of cases compared with controls with adequate coverage of the site differed by >10%. This site-based pruning resulted in exclusion of 4.8% of the Refseq coding sequence sites. We also introduced a likely gene-disrupting (LGD) model [23] to prioritize the candidate variants. The LGD model is defined by clustering LoF variants (nonsense, splice-site, and insertion/deletion). A damaging missense (D-mis) model was also applied for variant prioritization. D-mis is defined by selection of D-mis variants with a predicted CADD score ≥ 20. In viewing of the dominant traits that *FBN1* may have, inclusion criteria were strictly set to select the presumably LGD and D-mis variants to identify risk-conferring variants to CS. Variants that are not present at this time in 1KG, ESP, ExAC, dbSNP, the Universal Mutation Database for *FBN1* (UMD-FBN1; http://www.umd.be/FBN1/) [24] were defined as “novel.” Only novel variants were subjected to the burden analysis. We applied a collapsing method [25] to detect the association of mutational burden. The CADD score of 20, which corresponds to the top 1% of damage when evaluating all known allelic variants [26], was set as the cutoff value for creating a stratified variants subgroup for collapsing.

### Construction of expression plasmids

We constructed a plasmid expressing full-length *FBN1* (GenBank: NM_000138.4) cDNA with enhanced green fluorescent protein (EGFP) fusion, pEGFP-FBN1. A full-length *FBN1* cDNA having suitable restriction sites was PCR-amplified using KOD-Plus-Neo (Toyobo, Japan). The PCR amplicons were cloned into the *Nhe*I and *Sac*II sites of the pEGFP-N1 expression vector (Clontech, Takara Bio, Japan). The construct for the Leu871Phe variant was generated by QuikChange Lightning Site-Directed Mutagenesis Kit (Agilent Technologies, CA, USA). All plasmid constructs were subjected to Sanger sequencing for verification.

### Cell culture and transfection

HEK293T cells were cultured in Dulbecco's Modified Eagle Medium (DMEM) (Gibco, Waltham, MA, USA) supplemented with 10% FCS (Biological Industries, CT, USA) and 1% penicillin/streptomycin (Gibco) at 37 °C with 5% CO_2_. Cells were seeded in a six-well plates and transfected with Lipofectamine™ 3000 Transfection Reagent (Invitrogen, CA, USA) according to the manufacturers’ instructions. The medium was replaced with fresh DMEM culture medium 6 h after transfection. The cells were further incubated for 48 h.

### Real-time quantitative polymerase chain reaction

Total RNA was isolated using an RNeasy^®^ Mini Kit (Qiagen) and treated with RNase-Free DNase Set (Qiagen). Total RNA was reversely transcribed to cDNA by using the PrimeScript™ RT reagent Kit (#RR037A, Clontech). The relative abundance of targeted mRNA was normalized to glyceraldehyde-3-phosphate dehydrogenase (*GAPDH*). The primers 5’-CAAGGGCATCCTGGGCTACACT-3′ and 5′- CTCTCTCTTCCTCTTGTGCTCTTGC-3′ were used for amplification of *GAPDH*; 5′-ACCTGGTTACTTCCGCATAG-3′ and 5′-GAGGCATCAGTTTCGTTTGT-3′ were used for amplification of EGFP-FBN1. Gene expression was determined using SYBR Green PCR Master Mix (Applied Biosystems, CA, USA) on the 7500Fast Real-Time PCR Systems (Applied Biosystems).

### Western blot analysis

Cells were lysed with modified RIPA (50 mM Tris-HCL, 1% NP40, 0.25% Na-deoxycholate, 150 mM NaCl, and 1 mM EDTA; Complete^TM^ Protease Inhibitor Cocktail [Roche, Mannheim, Germany]), and protein concentrations were determined with the Pierce^TM^ BCA Protein Assay Kit (Pierce Biotechnology, Rockford, USA). A total of 5 mg protein was size separated on an 8% SDS polyacrylamide gel, and proteins were electrophoresed and transferred to nitrocellulose membranes. Membranes were blocked in powdered milk for 30 min at room temperature (RT, 25 °C), and primary antibodies (Phospho-Smad2 [Ser465/467] (138D4) Rabbit mAb #3108, Cell Signaling Technology, MA, USA; Smad2/3 [D7G7] XP^®^ Rabbit mAb #8685, Cell Signaling Technology; Mouse Anti-β actin mAb, TA-09, ZSGB-bio, China) were incubated overnight at 4 °C. After washing, the corresponding horseradish-peroxidase-coupled IRDye^®^ 800CW Goat anti-Mouse IgG Secondary Antibody (926-32210, LI-COR, NE, USA) were incubated for 1 h at RT. Bands were visualized with the WesternBright ECL HRP substrate chemiluminescent system (Advansta, CA, USA). Chemiluminescent signals were quantified using ImageJ [27].

### Statistical analyses

A one-sided Fisher’s exact test or a one-sided Chi-square test was used for all mutational burden analyses by using SPSS Statistics 23.0 (SPSS Inc., Al Monk, NY, USA), and a Student's *t* test was used to compare the differences of qPCR and WB results. All cell experiments were independently repeated three times with different cell lysates for each single assay, and data were presented as mean ± S.E.M. A *P* value < 0.05 was considered statistically significant.

## Results

### Identification of a novel *FBN1* null variant transmitted in a CS family

**Fig. 1:**
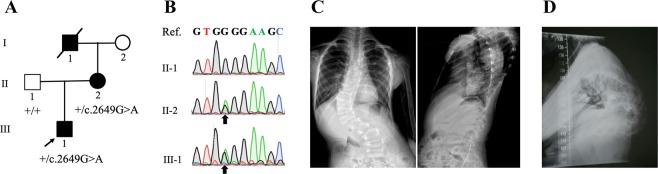
The family with congenital scoliosis and an *FBN1* nonsense variant, c.2649G>A (p.Trp883Ter). **a** Pedigree and segregation of the variant. **b** Electropherograms of Sanger sequencing showing the heterozygous c.2649G>A in the proband (III-1) and his mother (II-2). Ref., the reference sequence. Black arrows indicate the nucleotide substitution positions. **c** Anteroposterior and lateral X-ray film of the whole spine of the proband III-1 displaying congenital scoliosis with segmentation defects of thoracic 10-lumbar 1 spine with formation of vertebral bar on the left. **d** Lateral X-ray film of the whole spine of II-2 showing severe congenital kyphosis with segmentation defects.

We applied ES to identify responsible variants with autosomal dominant inheritance in all 103 familial CS. We identified a maternally inherited LGD variant associated with the vertebral phenotypes in a family. The novel heterozygous LGD variant was c.2649G>A (p.Trp883Ter) in *FBN1*, which co-segregated with CS in a dominant family (Fig. 1a, b). The nonsense variant was considered to exert a significant LoF effect by triggering the nonsense-mediated mRNA decay mechanism and subsequently loss of FBN1 protein. The proband III-1 displayed CS with structural segmentation defect of the vertebrae (T10-L1) and the formation of vertebral bar on the left side (T10-L1) (Fig. 1c), whereas the mother II-2 exhibited severe congenital kyphosis with segmentation defects (Fig. 1d). Although the mitral valve prolapse with mild mitral insufficiency was found in the proband, no other apparent cardiovascular or ocular phenotypes featuring MFS were identified in the proband and his mother. The aortic root measurements of the proband still remained normal (*Z* score = 1.4) by echocardiographic measurements, and the diagnosis of MFS could not be implemented according to the Ghent Criteria [28]. As for now, no obvious “marfanoid” appearances are observed in the family members. i.e., III-1 and II-2 (Supplementary Table 1).

In addition, we did not observe any LoF variants other than the *FBN1* p.Trp883Ter in any of the family members.

### Novel deleterious coding variants in *FBN1* are enriched in CS patients

Concerning the potential association between *FBN1* and CS, we further compared the difference of the frequency of *FBN1* variants between sporadic CS cases and controls. We subsequently identified a total of 30 novel variants (28 missense, 1 frameshift, and 1 splicing variant) in 16 out of the 574 CS cases and 14 out of 828 in-house controls. When all 30 variants were evaluated by the collapsing method, no significant association with CS was observed (OR = 1.6, *P* = 0.15 by one-sided Chi-square test). Next, the variants were stratified based on their deleteriousness determined by the CADD score. In the group of CADD score < 20, a total of 19 variants were subjected to the collapsed analysis without mutation types stratification and no statistical difference was identified. Further stratification within this group based on mutation types did not show any statistical difference in any mutation types (Table 1). In contrast, in the group of CADD score ≥ 20, a total of 11 missense variants were identified; eight in CS and three in controls, respectively. The eight missense variants (including one recurrent variant) were significantly enriched in CS (OR = 3.9, *P* = 0.03 by one-sided Fisher’s exact test) (Table 1). Sanger sequencing results of the eight cases are shown in Supplementary Fig. 1. Intriguingly, we have also checked the *FBN1* mutations in our study but did not identify any overlapping variants with the idiopathic scoliosis cohort reported by Buchan et al. [29].

**Table 1:** Mutational burden analysis of *FBN1* variants in congenital scoliosis.

Variant	Total number of variants	Number of alternative alleles		OR (95% CI)	*P* value
		Congenital scoliosis (*n* = 574)	In-house control (*n* = 828)		
Total	30	16	14	1.6 (0.8–3.2)	0.15
CADD score < 20	19	8	11	1.0 (0.4–2.6)	0.55
Frameshift	1	0	1	–	0.60
Splicing	1	1	0	–	0.41
Missense	17	7	10	1.0 (0.4–2.7)	0.60
CADD score ≥ 20	11	8	3	3.9 (1.0–14.6)	0.03
Missense	11	8	3	3.9 (1.0–14.6)	0.03

Association in the allelic model was calculated using Fisher’s exact test. *P* value < 0.05 was considered statistically significant

*OR* odds ratio, *CI* confidence interval, *CADD* Combined Annotation Dependent Depletion

### Genetic and phenotypic analyses of the CS individuals harboring candidate variants

**Fig. 2:**
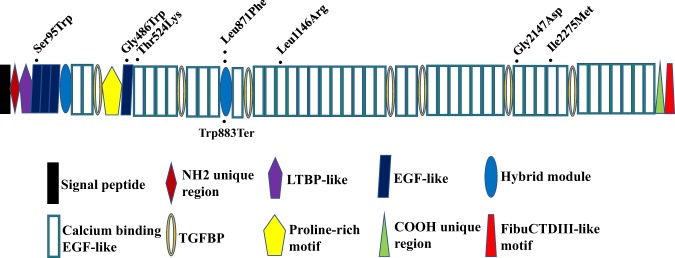
Schematic representation of the distribution of the candidate *FBN1* variants identified in congenital scoliosis. Protein structure is redrawn from the UMD-FBN1 database. Black dots indicate the location of missense and nonsense variants.

The genetic information and in silico deleteriousness predictions of the eight variants are presented in Table 2. One missense variant: p.Leu871Phe (CADD score = 23.9) was recurrent in two unrelated CS subjects. Leu871 was located within the hybrid 2 motif region of the protein (Fig. 2), which is an evolutionary ‘hybrid’ between TGF-β binding protein-like (TB) and calcium-binding epidermal growth factor-like (cbEGF-like) domains [30]. Previous studies indicated that mutations situated within this domain disrupted the ratio of an α-helix to β-sheet leading to a more compact conformation [31]. In addition, conservation analysis of amino acid residue of Leu871 in *FBN1* indicated that it is highly conserved throughout evolution and across many selected species, suggesting it is required for the normal function of the protein (Fig. S2). The remaining six missense variants predicted with highly deleterious properties were located in epidermal growth factor-like (EGF-like) domains or cbEGF-like domains (Fig. 2), playing a seminal role in diseases [32].

**Table 2:** Candidate *FBN1* variants in congenital scoliosis.

Subject ID	Genomic position^a^	Nucleotide change^b^	Protein change^c^	Exon number	Family and domain	AF in gnomAD		In silico prediction tool^d^			
						ALL	EAS	SIFT	Polyphen-2	Mutation taster	CADD score
XH1162	48902987	c.284C>G	p.Ser95Trp	4	EGF-like^e^ #01	0	0	D	D	D	22.7
XH810	48807596	c.1456G>T	p.Gly486Trp	12	EGF-like #04	0	0	D	D	D	24.6
XH152	48805763	c.1571C>A	p.Thr524Lys	13	cbEGF-likef #03	0	0	T	D	D	32
XH73	48787384	c.2613A>C	p.Leu871Phe	22	Hybrid motif #02	0.00000406	0.000058	D	D	D	23.9
XH579	48787384	c.2613A>C	p.Leu871Phe	22	Hybrid motif #02	0.00000406	0.000058	D	D	D	23.9
XH766	48779535	c.3437T>G	p.Leu1146Arg	28	cbEGF-like #13	0	0	D	P	D	26.4
XH902	48729214	c.6440G>A	p.Gly2147Asp	53	cbEGF-like #32	0	0	T	D	D	34
XH441	48722914	c.6825C>G	p.Ile2275Met	56	cbEGF-like #35	0	0	T	D	D	20.6

*gnomAD* Genome Aggregation Database, *AF* allele frequency, *ALL* all populations, *EAS* east Asian, *D* damaging, *P* probably damaging, *T* tolerated

^a^Chromosomal position based on GRCh37/hg19

^b,c^The RefSeq transcript number of *FBN1* is NM_000138.4

^d^In silico prediction tools to evaluate a genetic variant for its deleterious potential

^e^Epidermal growth factor-like domain

^f^Calcium-binding epidermal growth factor-like domain

The eight subjects harbored the *FBN1* variants displayed different types of vertebral malformations (Table 3). Malformations of the ribs were also found in three subjects (Table 3). Notably, intraspinal defects (diastematomyelia, syringomyelia, and tethered cord) were identified in five subjects (Table 3). Although mild mitral insufficiency and pectus carinatum deformity presented in XH902, aortic root measurements still remained normal (*Z* score = 1.6), and ocular phenotypes supporting MFS were not present. This patient would be categorized as “potential MFS” until the aorta reaches threshold (*Z* score = 3) [28]. The remaining seven subjects had no cardiovascular or ocular manifestations (Table 3), and hence they were not diagnosed as MFS. We have also re-evaluated the other marfanoid musculoskeletal phenotypes of these eight cases, and observed that no apparent manifestations except Patient XH902 with pectus carinatum deformity (Supplementary Table 2).

**Table 3:** Phenotypic manifestations of the congenital scoliosis subjects with deleterious *FBN1* missense variants.

Subject ID	Age (year)	Gender	MCA (degree)	Malformation involved					Other complication
				Vertebral	Costal	Intraspinal	Cardiovascular	Ocular	
XH1162	6	M	65	SD, FL, LF, H	N	N	N	N	Short neck
XH810	7	F	48	B	N	Tethered cord	N	N	N
XH152	11	M	72	H, W, FL	N	N	N	N	Pulmonary dysfunction
XH73	12	F	40	SD	Intercostal cohesion of bilateral 2–5th ribs	Diastematomyelia, hypoplastic spinal processes	N	N	N
XH579	2	M	104	W, FF, SD	Bilateral 12th rib absence	syringomyelia, tethered cord	N	N	Joint contractures
XH766	1	M	80	B, H	N	N	N	N	N
XH902	11	M	77	SD, W	N	Syringomyelia	Mitral insufficiency	N	Pectus carinatum
XH441	1	M	50	SD, FF, B	Bilateral 13th ribs	Diastematomyelia, syringomyelia	N	N	N

*MCA* maximal Cobb angle, *M* male, *F* female, *SD* segmentation defect, *FL* fused lamina, *LF* lamina fissure, *H* hemivertebrae, *B* butterfly-vertebrae, *W* wedge-shaped vertebrae, *FF* formation failure of vertebrae, *N* not present

### Functional study of the recurrent variant, Leu871Phe

**Fig. 3:**
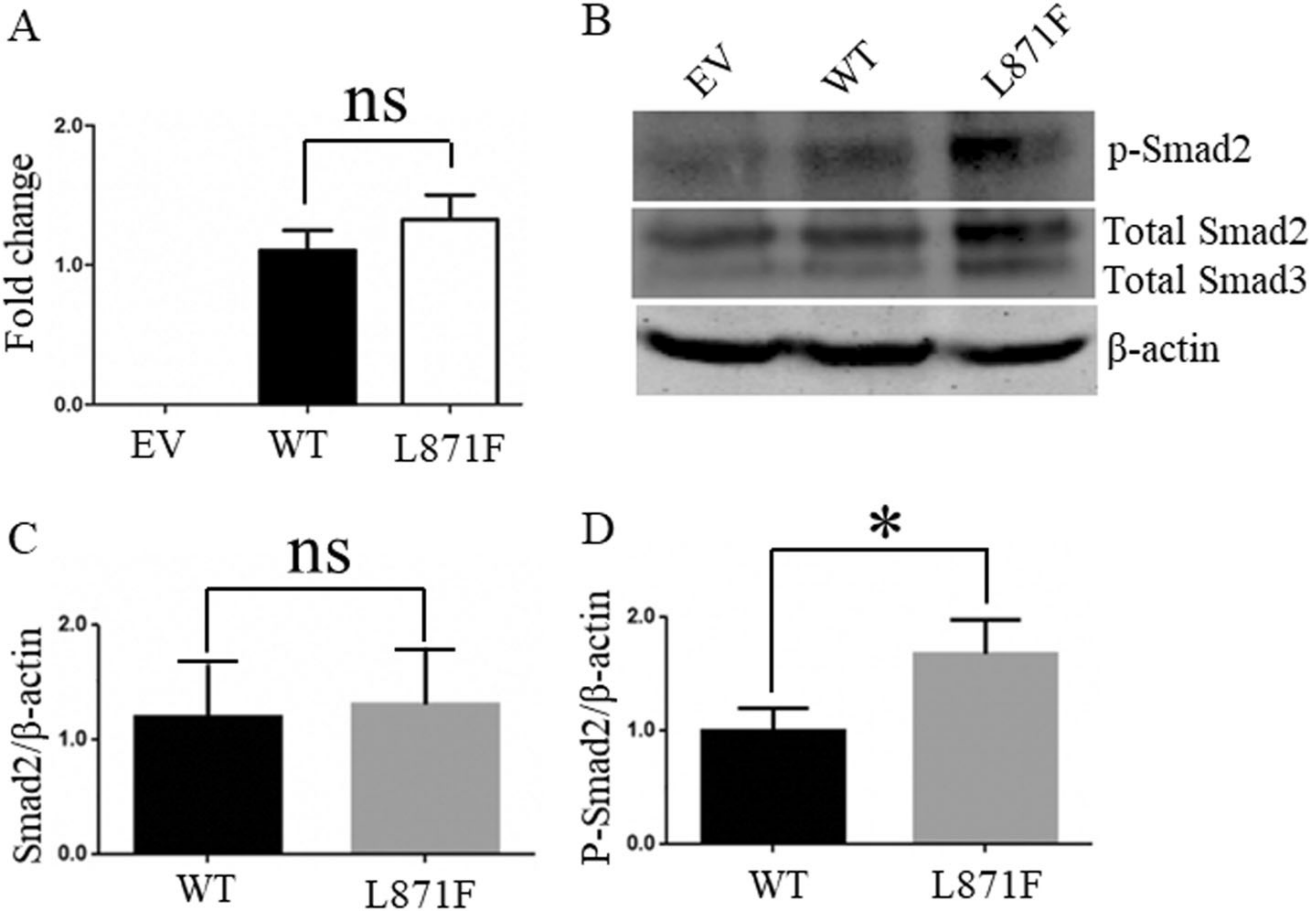
Functional analyses of the recurrent missense variant, c.2613A>C (p.Leu871Phe). **a** The expression of *FBN1* mRNAs from HEK293T cells transfected with the empty vector (EV), wild type (WT), or mutant (p. Leu871Phe: L871F) *FBN1* plasmids. ns not significant. **b** Western blotting for the protein lysates. **c, d** Quantification of the western blotting results. The ratio of phosphorylated Smad2 and total Smad2 to β-actin are shown normalized to WT plasmid. Data are expressed as arbitrary units indicating mean ± SEM of three independent experiments. Statistical significance was calculated by Student’s *t* test, *P < 0.05. ns not significant.

We overexpressed either human *FBN1* wild-type or p.Leu871Phe in HEK293T cells. Total RNA was reversely transcribed to cDNA 48 h after transfection. The mRNA expression level of the p.Leu871Phe FBN1 showed no statistical difference to that of the WT plasmid (*P* = 0.38 by Student’s *t* test) (Fig. 3a).

Previous studies have demonstrated that deleterious *FBN1* mutations upregulate endogenous TGF-β signaling, which could be measured via phosphorylation of Smad2 (p-Smad2), a downstream target [33]. Transfected cells with the p.Leu871Phe FBN1 showed significantly elevated p-Smad2 level compared with the WT (*P* = 0.02 by Student’s *t* test) (Fig. 3b–d), while they showed no statistical difference in expression of total Smad2 compared with the WT (*P* = 0.96 by Student’s *t* test).

## Discussion

In the present study, we conducted family-based ES analysis and allele-based mutational burden analysis, and provided evidence for the genetic association between *FBN1* and CS. We then performed in vitro functional assay for a recurrent *FBN1* variant (p.Leu871Phe) and revealed the upregulation of TGF-β signaling caused by the variant. Clinical features for the CS cases with *FBN1* variants showed the expansion of the *FBN1-*related disease entity.

*FBN1* encodes a connective tissue protein essential to extracellular microfibrils organization in dermal fibroblasts and skeletal muscle cells [34]. *FBN1* is well known as a causal gene for MFS, which often has scoliosis without structural changes of the vertebrae and ribs. On the other hand, *FBN1* mutations also cause various skeletal dysplasia, including Acromicric dysplasia (MIM #102370), Geleophysic dysplasia 2 (MIM #614185), Marfan lipodystrophy syndrome (MIM #616914), and Weill–Marchesani syndrome 2 (MIM #608328). These monogenic diseases have a broad range of vertebral phenotypes quite different from MFS. In a previous study, a novel *FBN1* variant, p.Gly1796Glu co-segregated in a family with an autosomal dominant vertebral dysplasia featuring CS, in the absence of cardiac or ocular findings [35], indicating that *FBN1* could be a disease gene for monogenic form of CS. Our case also confirmed that *FBN1* causes CS as an autosomal dominant disease trait pattern.

The index case in our study was from an unusual three-generation CS family segregating *FBN1* LoF variant, c.2649G>A (p.Trp883Ter), which has been previously reported to be a pathogenic nonsense mutation associated with MFS [36], and deposited in HGMD (accession ID: CM161831). This nonsense substitution truncates the protein at codon 883, which is 1989 amino acids from the end of the protein. According to the ACMG guidelines for classifying pathogenic variants [37], this nonsense mutation is a convincing pathogenic LoF allele based on the following evidences: (a) PVS1: this nonsense variant is interpreted at the internal part of *FBN1* gene where LoF is a known mechanism [38]; (b) PP1: cosegregation with CS phenotypes in the affected family members; (c)PP3: multiple computational evidence supports highly deleterious properties (Mutation Taster prediction shows deleterious; conservation prediction score by GERP is 4.81; CADD score is 42); (d)PP5: Franken et al. recently reported this variant as a pathogenic LoF allele associated with MFS, but the detailed evaluations for spinal/vertebral phenotypes were unavailable [36]. Notably, it has been widely reported that the high degree of clinical variability associated with *FBN1* variants. For instance, distinct genetic mechanisms, encompassing a second deleterious variant in another gene or a polygenic model involving modifier loci, are proposed to account for this clinical variability [39]. Thus, the occurrence of a pathogenic variant in *FBN1* is not necessarily associated with or produces MFS. Consistently with our findings, some *FBN1* nonsense mutations (c.284C>A, p.Ser95Ter; c.1347_1348dupTA, p.Thr450IlefsTer130) only caused isolated skeletal features, with no cardiac or ocular findings [40]. Pathogenic *FBN1* variants cause MFS but can also be found in patients presenting with apparently isolated features. For instance, the c.1453C>T, p.(Arg485Cys) mutation in *FBN1* has been identified in both autosomal dominant and recessive diseases characterized by a high extent of phenotypic variability [41, 42]. Hence, it is reasonable to postulate that the c.2649G>A allele, which has been associated with MFS, could also account for the isolated CS in our study.

*FBN1* is implicated in vertebral growth and development [43]. Fibrillin microfibrils in the calf are ubiquitously distributed in the vertebral growth plate and involved in growth factor binding, playing a pivotal role in regulating bone growth [43]. Moreover, a murine model with an intragenic duplication of *Fbn1* presented markedly enlarged lumbar vertebra and elongated costal cartilages [44]. Functional follow-up of *Fbn1*^Tsk^ mouse unveiled the interactions of *FBN1* with the Wnt signaling pathway [45] and its pivotal role for somitegenesis and vertebrate segmentation [4]. Since CS is induced by localized changes in vertebral body development [46], we proposed that factors distorting the normal localization process of the spine column may contribute to the pathogenesis of CS, such as *FBN1*.

TGF-β plays critical roles in development and maintenance of the skeleton [47]. The two unrelated patients (XH73 and XH579) sharing the p.Leu871Phe variant presented CS with vertebral segmentation defects, rib anomalies, and diastematomyelia. Our in vitro experimental results showed upregulation of the TGF-β signaling by the p.Leu871Phe variant, supporting the pathogenicity of the mutation. TGF-β signaling plays a critical role in cartilage and spine tissue development, upregulation of the TGF-β signaling pathway within the intervertebral disc could cause the occurrence of vertebral fusions [48]. The observations suggest that the upregulation of the TGF-β signaling pathway confer susceptibility to CS. In prior reports, the excess TGF-β activation and signaling have been frequently observed in *FBN1*-deficient humans and mice [29, 49].

Regarding a mutational burden test for CS with a complex disease trait, the most important thing is not to focus disproportionately on specific variants, but rather to integrate across all types of risk-associated variants. In some individuals, risk may be caused by an unusual combination of common variants and rare variants, like the TACS compound inheritance model [6–8], whereas in others it may be due to an accumulation of rare or ultra-rare variants. In this context, it seemed actionable to stratify the *FBN1* variants by the mutation types and CADD C-score prior to detecting the association by the collapsing method [25]. The knowledge about the variants associated with CS and the variable genetic penetrance will blaze the trail for genetic counseling and evaluating of the relatives of the variant carriers.

In conclusion, our observations indicated that *FBN1* may be a susceptibility gene for CS, expanding our knowledge to the existing list of predisposition and candidate genes in CS phenotypes. It remains unknown why distinct mutations in the *FBN1* gene can convey a variable continuum of skeletal disorders, ranging from the overlapping features to discordant phenotypes. Future studies focusing on exploring the underlying mechanisms of pleiotropic effects of *FBN1* may be warranted.

## Supplementary information

Table S1

Table S2

Figure S1

Figure S2
