# Supplementary material for: Identification of novel FBN1 variations implicated in congenital scoliosis
Source: J Hum Genet. 2019 Dec 11;65(3):221–30. doi: 10.1038/s10038-019-0698-x (PMC6983459; doi:10.1038/s10038-019-0698-x)
Supplement: Supplementary file 1 — Table S1 [file 10038_2019_698_MOESM1_ESM.docx]

**Table S1** Evaluation of musculoskeletal phenotypes of III-1 and II-2 in this CS family

| Musculoskeletal phenotypes | III-1 | II-2 |
| --- | --- | --- |
| Wrist/thumb sign | A | A |
| Arachnodactyly | A | A |
| Pectus carinatum/excavatum | A | A |
| Joint laxity | A | A |
| Arm span to height | 0.96 | 0.90 |
| Upper/lower segment | 0.92 | 0.94 |
| Skin striae | A | A |

A denotes absent.
