# Supplementary material for: Identification of novel FBN1 variations implicated in congenital scoliosis
Source: J Hum Genet. 2019 Dec 11;65(3):221–30. doi: 10.1038/s10038-019-0698-x (PMC6983459; doi:10.1038/s10038-019-0698-x)
Supplement: Supplementary file 2 — Table S2 [file 10038_2019_698_MOESM2_ESM.docx]

**Table S2** Evaluation of musculoskeletal phenotypes in these 8 CS cases

| Musculoskeletal phenotypes | XH1162 | XH810 | XH152 | XH73 | XH579 | XH766 | XH902 | XH441 |
| --- | --- | --- | --- | --- | --- | --- | --- | --- |
| Wrist/thumb sign | A | A | A | A | A | A | A | A |
| Arachnodactyly | A | A | A | A | A | A | A | A |
| Pectus carinatum/excavatum | A | A | A | A | A | A | P | A |
| Joint laxity | A | A | A | A | A | A | A | A |
| Arm span to height | 0.88 | 0.86 | 0.84 | 0.92 | 0.85 | 0.83 | 1.00 | 0.90 |
| Upper/lower segment | 0.93 | 0.82 | 0.85 | 0.94 | 0.90 | 1.10 | 0.98 | 0.96 |
| Skin striae | A | A | A | A | A | A | A | A |

P denotes present; A denotes absent.
