# Supplementary material for: Identification of novel FBN1 variations implicated in congenital scoliosis
Source: J Hum Genet. 2019 Dec 11;65(3):221–30. doi: 10.1038/s10038-019-0698-x (PMC6983459; doi:10.1038/s10038-019-0698-x)
Supplement: Supplementary file 3 — Figure S1 [file 10038_2019_698_MOESM3_ESM.pptx]

## Slide 1
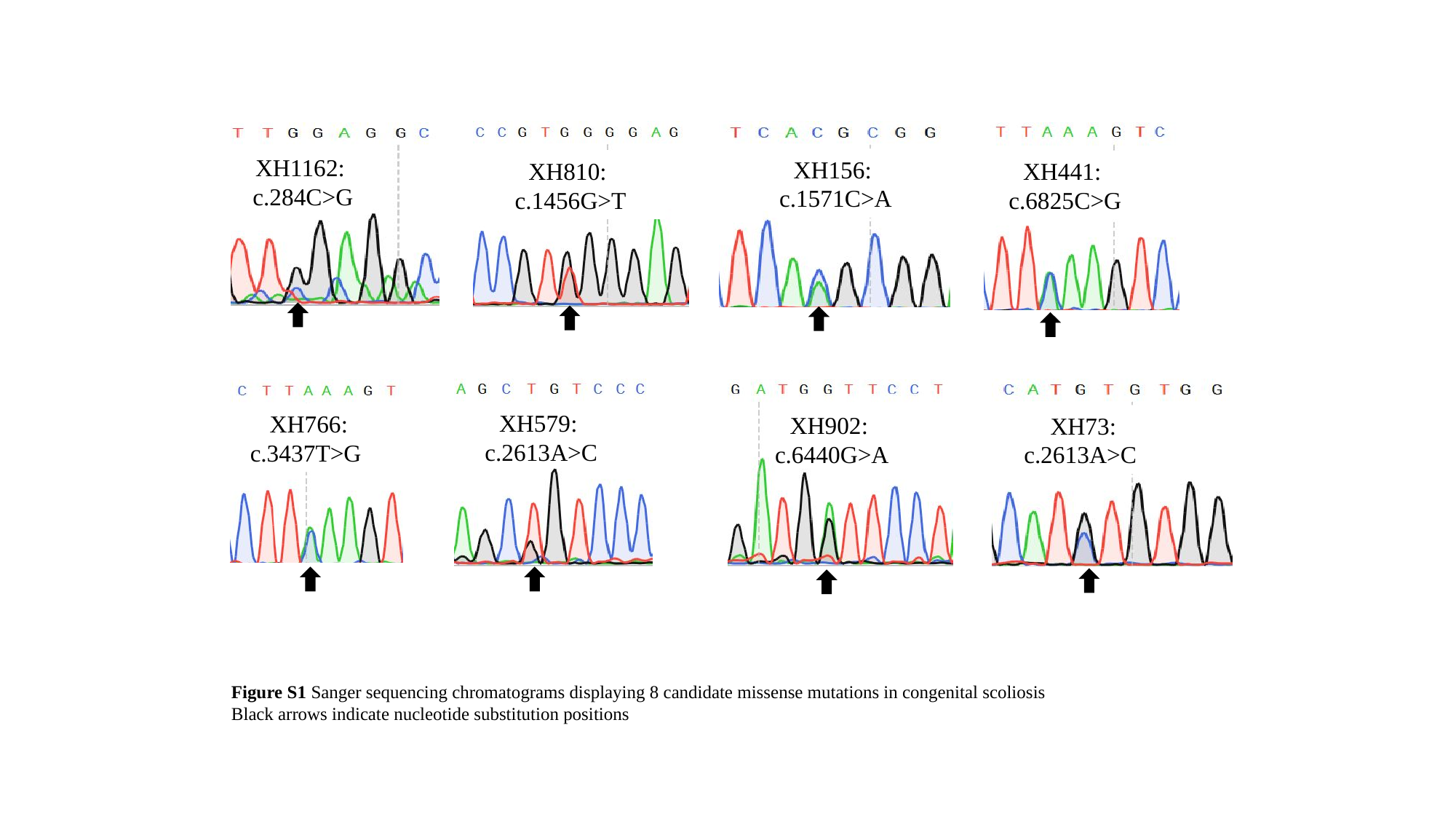

XH1162:
c.284C>G
XH156:
c.1571C>A
XH810:
c.1456G>T
XH441:
c.6825C>G
XH579:
c.2613A>C
 XH766:
c.3437T>G
XH902:
c.6440G>A
 XH73:
c.2613A>C
Figure S1 Sanger sequencing chromatograms displaying 8 candidate missense mutations in congenital scoliosis
Black arrows indicate nucleotide substitution positions
