# Supplementary material for: Identification of novel FBN1 variations implicated in congenital scoliosis
Source: J Hum Genet. 2019 Dec 11;65(3):221–30. doi: 10.1038/s10038-019-0698-x (PMC6983459; doi:10.1038/s10038-019-0698-x)
Supplement: Supplementary file 4 — Figure S2 [file 10038_2019_698_MOESM4_ESM.pptx]

## Slide 1
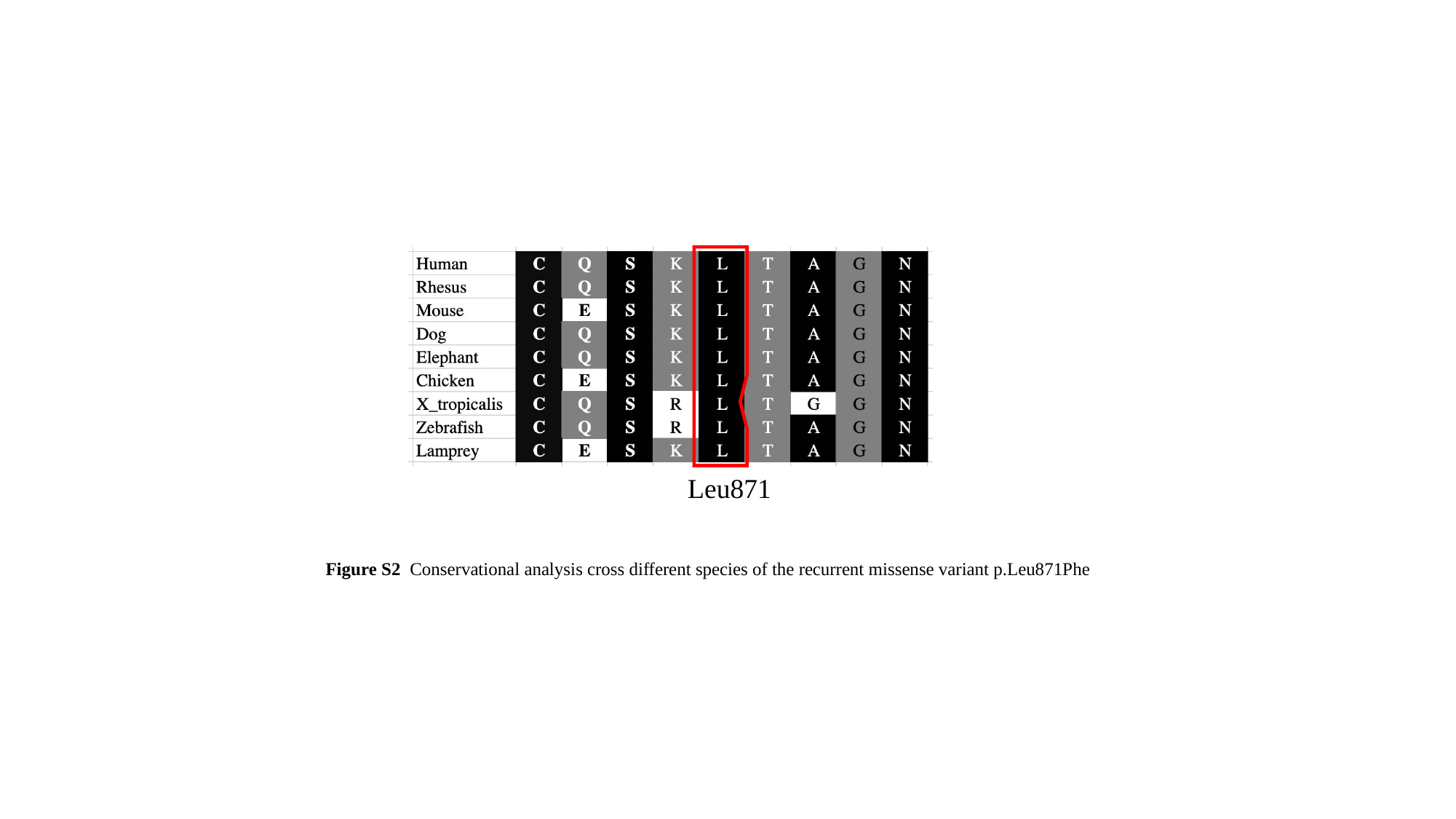

Leu871
Figure S2 Conservational analysis cross different species of the recurrent missense variant p.Leu871Phe
